# Immunopathological Mechanisms Underlying Cardiac Damage in Chagas Disease

**DOI:** 10.3390/pathogens12020335

**Published:** 2023-02-16

**Authors:** Mariana Citlalli De Alba-Alvarado, Elia Torres-Gutiérrez, Olivia Alicia Reynoso-Ducoing, Edgar Zenteno-Galindo, Margarita Cabrera-Bravo, Yolanda Guevara-Gómez, Paz María Salazar-Schettino, Norma Rivera-Fernández, Martha Irene Bucio-Torres

**Affiliations:** 1 Departamento de Microbiología y Parasitología, Facultad de Medicina, Universidad Nacional Autónoma de México, Coyoacán, México City 04510, Mexico; 2 Departamento de Bioquímica, Facultad de Medicina, Universidad Nacional Autónoma de México, Coyoacán, México City 04510, Mexico

**Keywords:** immunopathology, zoonotic disease, *Trypanosoma cruzi*, Chagas disease, cardiopathy

## Abstract

In Chagas disease, the mechanisms involved in cardiac damage are an active field of study. The factors underlying the evolution of lesions following infection by *Trypanosoma cruzi* and, in some cases, the persistence of its antigens and the host response, with the ensuing development of clinically observable cardiac damage, are analyzed in this review.

## 1. Introduction

Chagas disease is a complex zoonosis. Its natural history involves the interaction of transmitting arthropods with wild, peridomestic, and domestic mammals, and it has a great diversity of transmission forms. In a vertebrate host, the disease evolution is evinced by various clinical manifestations [1,2].

The disease is caused by *Trypanosoma cruzi*, a flagellated protozoan that is naturally transmitted by hematophagous Hemiptera insects (triatomines). The parasite was discovered in 1909 by Dr. Carlos Chagas in Minas Gerais, Brazil. Dr. Chagas also described a large part of the biological cycle, linking the parasite to the transmitting triatomine (*Panstrongylus megistus*). He was able to isolate the parasite and replicated the infection in experimental animals. Dr. Chagas also mentioned that rural housing conditions, which to date have not changed significantly in endemic countries, are important for the spread of the vector. As a clinical entity, Chagas disease is linked to poverty [1].

Chagas disease is endemic in 21 countries within continental Latin America. It is distributed from the south of the United States, in Central America, the Southern Cone, Andean countries, and Amazonian countries. In the Americas, 30,000 new cases are reported every year, 12,000 deaths on average, and 8600 newborns are infected at gestation. In 2019, a prevalence rate of 933.76 per 100,000 population was recorded. Currently, about 70 million people in the Americas live in areas at risk for the infection [3]. Humans become infected with *T. cruzi* by several mechanisms. The most important one is natural transmission, involving an infected triatomine bug. This transmission form is very common in rural areas, where housing traits and the ecotope favor a colonization of the domestic niche by insects. The second transmission form, restricted to urban areas, is related to the transfusion of blood or its components. This transmission form depends on the migration of rural population to cities, as more than 70% of the population in some cities had immigrated from high-prevalence areas of the disease [4].

The primary vectors for Chagas Disease are *Triatoma infestans* in Argentina, Bolivia, Brazil, Chile, Paraguay, Uruguay, and Peru; *Rhodnius prolixus* in Colombia, Venezuela, and Central America; *T. dimidiata* in Ecuador and Central America; and *R. pallescens* in Panama [3]. In the Americas, natural infection is associated with risk factors such as housing construction material and other characteristics that favor the colonization of human dwellings in rural areas. For this reason, it is considered as a neglected tropical disease [4]. PAHO/WHO, working with the endemic countries, have launched several Subregional Disease Prevention and Control Initiatives. These include the improvement of housing to halt vectorial transmission in 17 countries, and screening of blood donors in the 21 endemic countries, in addition to eliminating some vector species such as *R. prolixus* in El Salvador, Costa Rica, and Mexico, and *T. infestans* in Brazil and Uruguay [3,5].

Due to the increase in population mobility worldwide, Chagas disease is considered a major health problem, which has reached countries where vector transmission does not exist, due to the immigration of seropositive cases from endemic geographic areas. This poses a risk of transmission through transfusions, organ and tissue transplants, and even maternal-fetal transmission in the USA, Canada, and some European and Western Pacific countries. It has been estimated that Spain is the main non-endemic country in the number of transmissions, followed by the USA and Italy [6].

Chagas disease has two clinical phases: an acute phase and a chronic one. Most cases of acute Chagas disease are asymptomatic; only 5–10% of infected subjects develop symptoms, including persistent fever, asthenia, adynamia, headache, and hepatosple-nomegaly, all of which are nonspecific. Among these symptomatic individuals, the most frequent pathognomonic signs are the Romaña sign, characterized by unilateral bi-palpebral edema, which is observed in 50% of cases, and the indurated cutaneous chagoma, found in 25% of cases. Both signs are often accompanied by regional adeno-megaly. In the remaining 25% of patients, there is no sign of portal entry, but some of the nonspecific symptoms mentioned above can be found [7]. The most frequent symptom is fever, which is present in up to 95% of cases, usually without specific characteristics. All other signs and symptoms, including asthenia, adynamia, headache, and hepatosple-nomegaly, are nonspecific [3,5]. While *T. cruzi* can infect any nucleated cell, some strains exhibit a marked tropism for myocardial cells, smooth muscle cells of the digestive system, or nervous tissue, among other cell types. Cardiac manifestations include primarily organ enlargement (cardiomegaly) [7].

The chronic phase is divided into a chronic asymptomatic phase and a symptomatic phase; the former evolves without demonstrated pathology and can last 10–20 years; however, cases have been reported in minors in Mexico where this period lasts 2–7 years before the chronic form with cardiovascular symptomatology can be detected [8]; this phase is clinically asymptomatic and exhibits very low parasitemia, so that the methods of choice for diagnosis are serological, and confirmation requires two positive tests with different principles [9].

After this phase, patients progress to the chronic symptomatic phase, with a proven symptomatology, in which they develop mainly cardiac lesions and, to a lesser extent, digestive lesions, mainly in the esophagus and colon, and in a few cases in the peripheral nervous system. Cardiac lesions cause alterations in myocardial contractility and the electrical impulse, mainly in the bundle of His, the particular right bundle branch block with left anterior fascicular hemiblock, ventricular extrasystoles, and atrioventricular block [3,7]. The lesions in this cardiomyopathy involve several cardiac tissues, mainly the myocardium, and in severe cases, the endocardium pericardium; this can cause pleural effusion, which may evolve into sudden death, which is more frequent in cases with dilated heart disease and severe heart failure [10]. Carlos Chagas described digestive disorders linked to the disease in 1916, although Kidder and Fletcher had already done so in 1857, when they called it mal d’engasgo, i.e., “disease causing dysphagia” [11]. Esophageal involvement usually consists of a megaesophagus with slow esophageal transit disorders, along with pain and difficulty in swallowing. In cases of colon involvement, a megacolon and constipation are typical [12,13]. Chagas heart disease is clinically classified according to symptoms, and electrocardio-graphic, echocardiographic, and radiological abnormalities, especially changes in left ventricular function. Some risk factors predisposing one to a progression to the chronic phase include electrocardiographic abnormalities, male sex, systolic blood pressure less than 120 mmHg, altered systolic function, left ventricular dilatation, and complex arrhythmias; risk scores have been proposed to stratify the risk of death, although their clinical value is still under study [14]. In a consensus, several authors note that the main predictors of poor prognosis in chronic Chagas disease are a deterioration of left ventricular function, falling into classes III (fatigue, palpitations, dyspnea, or anginal pain) and VI (cardiomegaly and non-sustained ventricular tachycardia) of the New York Heart Association (NYHA) classification of heart failure [15]. Other risk scores have been proposed. Rassi uses a combination of clinical symptoms, cabinet test results, and demographic data [16]. On the other hand, de Sousa use a four-factor score that includes the QT dispersion interval on ECG, syncope, premature ventricular contractions, and left ventricular function [17].

Benznidazole and nifurtimox are the only drugs with proven efficacy against Chagas disease. Both drugs have been approved internationally. Antiparasitic treatment of these cardiomyopathies is accompanied by the administration of antiarrhythmics and pace-maker placement. In the most severe cases, heart transplantation can be required [5,7].

## 2. Pathogenic Mechanisms of Immune Response Evasion in Chagas Disease

Most of the mechanisms involved in evading the immune response occur in the acute phase, when trypomastigotes establish contact with immune cells of the vertebrate host. The parasite has evolved mechanisms to survive processes such as phagocytosis and the complement system, in addition to interfering with lymphocyte maturation. When metacyclic trypomastigotes contact host cells, either through skin lesions or mucous membranes, the immune response is activated. *T. cruzi* enters cells through two main mechanisms. The first one is lysosome-dependent, which favors Ca^2+^ mobilization [18]. In this stage, the parasite surface glycoprotein gp82 is crucial for cell adhesion and lysosomal fusion at the site of entry. Cruzipain has also been proven to be critical for calcium induction and lysosome recruitment. It is a cysteine-protease secreted by trypomastigotes [19,20]. The second mechanism involves invagination of the plasma membrane followed by lysosomal fusion. The acidification process of lysosomes containing the parasite is key to its differentiation into an amastigote, which is the replicative form. A disruption of cytoskeletal actin facilitates the mobilization of lysosomes towards the cell periphery, where they will fuse with the cytoplasmic membrane and contribute to the formation of a parasitic vacuole [21]. After several divisions in the parasitophorous vacuole, where sialic acid residues are added on the *T. cruzi* membrane by parasitic trans-sialidases, the phagolysosome is lysed. Thus, trypomastigotes are released into the cytoplasm and bloodstream to infect distant or adjacent tissues and cells [22].

*T. cruzi* can also be phagocytosed at the site of infection by tissue macrophages. *Leishmania* spp. also parasitizes macrophages and develops mechanisms similar to those of *T. cruzi* [23]. Some *Leishmania* species that parasitize cutaneous and peripheral blood macrophages and have been isolated in Latin America are *L. mexicana*, *L. amazonensis*, *L. venezuelensis*, and *L. braziliensis*; in the Mediterranean, we find *L. infantum*, and in Asia there are *L. tropica* and *L. major* [23]. It should be emphasized that these species, phylogenetically related to *T. cruzi* as Kinetoplastida, can inhibit the antiparasitic function of macrophages, and both genera use this strategy; in the case of *Leishmania* spp., they create a safe intracellular compartment and continue their life cycle in the mammal, and in the case of *T. cruzi*, they evade the phagolysosome and escape to the cytosol for replication [24,25]. To survive in this extremely oxidative environment inside the macrophages, the *T.cruzi* express antioxidant enzymes such as peroxidases, which protect it from reactive oxygen and nitrogen species within macrophages [26,27]. In this regard, an overexpression of TcCPX in *T. cruzi* has been shown to correlate with increased parasitemia and inflammatory infiltrates in the myocardium [28].

Notably, factors such as strain, the level of antioxidant enzyme expression, and the kinetics of association with the phagolysosome may in turn contribute to parasite evasion and persistence in the host. In contrast to other protozoa, which inhibit phagolysosome maturation, *T. cruzi* evade macrophage activity by the mechanisms mentioned above, and escape from the phagolysosome into the host cell cytoplasm, where they replicate [29]. Once outside the macrophages or in any infected tissue, *T. cruzi* can be recognized by their different PAMPs, which are mainly glycoinositolphospholipids (GIPLs) and lipopepti-doglycans (LPPG). These molecules have protective functions because they allow the parasite to survive in hydrolytic environments and promote adherence to mammalian cells for invasion. The complement system features a specialized pathway for mannose recognition in pathogenic organisms, and it is known that blood trypomastigotes activate this system; however, the parasite expresses a set of specific surface proteins for complement evasion [30,31]. *T. cruzi* trans-sialidases are crucial for host cell infection by transferring sialic acid from mammalian cells to their own glycocalyx [32]. Their presence is also known to reduce the recognition of anti-α-Gal antibodies in the bloodstream, in addition to evading the lytic effect of complement by promoting the conversion of C3 to inactive C3b (iC3b) [33].

*T. cruzi* calreticulin (TcCRT) is also involved in the evasion of the lectin pathway by binding to MBL or inhibiting the classical complement pathway by binding to C1q. These links inactivate the membrane attack complex (MAC) formation pathway [34]. Another mechanism by which the parasite controls the complement pathway is mediated by the CRP protein, which is anchored to the parasite membrane. This 160-kDa glycoprotein binds non-covalently to C3b and C4b to inhibit the assembly of C3 convertase, rendering it inactive to catalyze the cleavage of the complement system on the parasite surface. Another parasitic protein called T-DAF accelerates the decay of C3 and C5 convertase in the classical and alternative pathways of the complement system. The mucin-rich surface of *T. cruzi* can be recognized by Toll-like receptors, such as TLR-2, expressed on mac-rophages. This interaction induces the synthesis of proinflammatory cytokines such as IL-12 and TNF-α, and it favors the activation of the iNOS pathway in these phagocytes. In addition, cruzipain from *T. cruzi* has a proteolytic action on the NF-κB protein complex, inhibiting the transcription of proinflammatory cytokines such as IL-12 [35].

## 3. Histopathological Mechanisms Related to Tissue Parasitism

**Figure 1:**
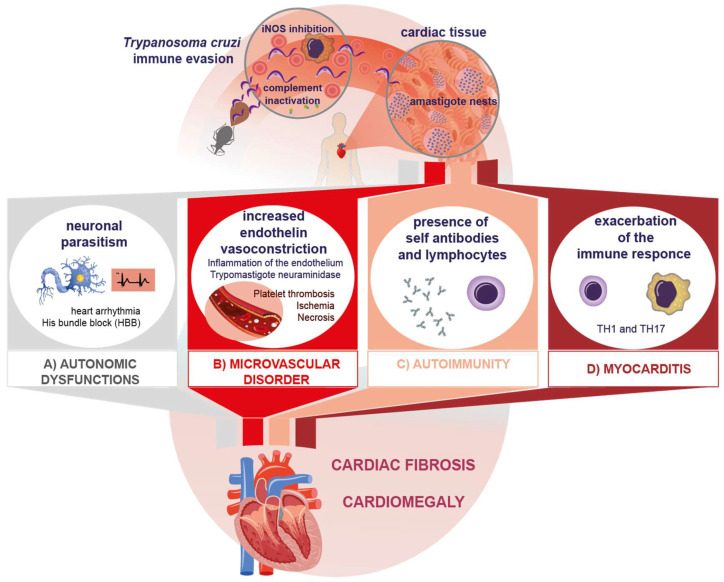
Immunopathological mechanisms of Chagas disease. The four immunopathogenic mechanisms interacting to cause fibrosis and cardiomegaly in the chronic phase of Chagas disease.

The presence and replication by binary fission of intracellular amastigotes in the myocardiocyte and its ensuing lysis cause inflammation, the release of cellular components, and finally the destruction of cardiac tissue [36]. A direct correlation between the presence of the parasite and tissue inflammation has been reported [37], even when only the presence of *T. cruzi* antigens or DNA has been confirmed in chronic lesions [38]. The infection can affect skeletal, cardiac, and smooth muscle, as well as neuronal tissue. Parasitism in the myocardium causes destruction and fibrosis in the Schwann sheath and nerve fibers, which leads to neurogenic alterations, clinically expressed as arrhythmias, His bundle branch block, and even the presence of dyssynergic areas in the parietal function of the heart (Figure 1A). All of these are characteristic manifestations of the chronic phase of Chagas disease [39,40]. Another important mechanism described in myocardial injury is the occurrence of microvascular abnormalities caused by the increase in endothelin due to inflammation. On the other hand, blood trypomastigotes have been described to produce neuraminidase (Figure 1B). All these mechanisms increase platelet adhesiveness and thrombus formation, leading to myocardial ischemia and microinfarcts, which in turn cause tissue necrosis [41,42].

As mentioned above, chronic myocardial inflammation and proinflammatory cytokines can affect myocardial microcirculation. Constant episodes of intense microcirculatory vasodilatation may occur, leading to decreased myocardial blood flow in the terminal distal portions of the coronary microcirculation, thus promoting myocardial ischemia of the apical infero/lateral and basal segments [43]. Rochitte et al. have observed this mechanism, determining by magnetic resonance imaging that the apical and inferolateral regions of the left ventricle show greater damage due to myocardial fibrosis [44]. This is consistent with experimental findings, where chagasic myocardial fibrosis is found in regions of terminal circulation, such as the apex (terminal circulation between the left anterior descending and right coronary artery) and the basal inferolateral segments (terminal circulation between the right coronary artery and the left circumflex coronary artery) [45]. As damage progresses, it leads to myocardial thinning, which results in hypokinesia of the cardiac wall, and eventually to apex aneurysms, a common finding in patients with severe Chagas disease [46].

## 4. Autoimmune Mechanisms in Chagas Disease

Several theories have been advanced on the development of lesions in heart disease. Among them, the presence of autoimmune phenomena and the persistence of the parasite stand out, without being mutually exclusive [47]. To date, there is no consensus regarding the autoimmune hypothesis in the pathogenesis of Chagas disease; however, several studies have demonstrated the participation of mechanisms that could contribute to tissue damage in the chronic phase [47,48]. Autoimmune damage processes can originate through several mechanisms: (1) In the acute phase, lysis of infected vertebrate cells leads to an exposure of cryptic antigens or with molecular mimicry between the parasite and host cell epitopes. (2) The release and presentation of self-antigens. The ensuing production of inflammatory cytokines may exceed the activation threshold, breaking self-tolerance and stimulating T and B lymphocytes [49]. (3) T cell activation without cytokine signaling (bystander activation). (4) Autoantibody-dependent cell-mediated cytotoxicity [50]. All four mechanisms may result in autoimmune damage.

The existence of molecular mimicry between proteins and other molecules that generate clones of autoreactive CD4+ and CD8+ T lymphocytes, which simultaneously recognize cardiac myosin and the B13 protein of the parasite, has been demonstrated [51]. The proteins with mimicry between host and *T. cruzi* antigens are shown in Table 1.

Other processes inherent to the presentation of host antigens after myocardial lysis suggest the existence of cryptic antigens. This is the case in traumatic ophthalmopathies, which expose previously cryptic antigens. Since tolerance for these antigens has not been developed, autoreactive clones are produced. Similarly, some cryptic epitopes can show molecular mimicry with *T. cruzi* epitopes, which favors the generation of these clones [64]. Such a process has been reported in Chagas disease after host cell lysis (Figure 1C).

As shown in Table 2, there is evidence of the generation of autoreactive T cells in Chagas disease. Ribeiro Dos Santos et al. described myocarditis induced by CD4+ T cell lines from mice in the chronic phase that proliferate in response to any crude *T. cruzi* antigen or heart tissue extracts [65]. The existence of T lymphocyte populations that recognize cardiac myosin in inflammatory infiltrates during the chronic phase has been reported in T cells in asymptomatic patients that respond to B13 antigen stimulation, which causes increased interferon-γ production. Cardiac damage could be due to delayed hypersensitivity, initiated by antigens such as B13, which induce the release of inflammatory cytokines [66].

The loss of tolerance of T lymphocytes could also be due to a defect in thymic reg-ulation of the formation of naïve T lymphocytes. Since *T. cruzi* cannot synthesize sialic acid, it expresses trans-sialidases that alter the maturation of thymocytes, causing the migration of undifferentiated cells to the circulation. In this regard, increased numbers of double positive TCD4+ and TCD8+ cells have been demonstrated in this infection [73].

## 5. Inflammatory and Cytotoxic Process in the Immunopathogenesis of Chagas Disease

The inflammatory process in Chagas disease starts in the acute phase, with the production of proinflammatory cytokines to recruit and induce the activation of monocytes to the site of infection. This process is important to control parasite replication through a Th1-type response, characterized by the production of interleukins (IL) such as IL-1, inter-feron gamma (IFN-γ), and the tumor necrosis factor alpha (TNF-α). The functions of IFN-γ predominate in this mechanism by inducing the production of nitric oxide (NO) in macrophages and initiating the control of blood parasitemia in this phase of the disease [74]. On the other hand, it has also been reported that IL-10 and IL-4, stimulated by antigens such as cruzipain, reduce the production of IFN-γ and NO in macrophages, inhibiting phagocytosis [75].

As noted, while the Th1 response protects the host in the acute phase, its deregulation in the chronic phase leads to cardiac tissue damage by exacerbated, chronic inflammation (Figure 1D) [76]. Molecules that contribute to tissue damage (DAMPS), specifically of cytotoxic origin, are generated by inflammation [77]. In this regard, there is evidence that oxidative stress also induces myocardial lesions in these patients, contributing to lesion progression in Chagas disease [78].

As the condition progresses towards chronicity, further mechanisms of cytotoxic damage affect cardiac tissue. Initially, oxidative stress is triggered, and reactive oxygen species are released. These reactive oxygen species, mainly H_2_O_2_ and OH, can be pro-duced by cytotoxic immune responses that cause lysis in the tissues surrounding the infec-tion [79,80]. Another mechanism triggered by the production of reactive oxygen species is the increase in NO levels, which stimulates the production of proinflammatory cytokines and the activation of adhesion molecules for rolling, as well as an increased endothelial permeability [81]. Immune cells with cytotoxic activity, such as macrophages and neutrophils, cause oxidative stress. The inflammatory infiltrate generated in the response against pathogens can subsequently cause myocardial damage. Neutrophils are the first cell type involved in this process and contribute to tissue damage. It has been described that, after infarct resolution, the presence of neutrophils either decreases rapidly, or they may remain activated if inflammation is not resolved. Neutrophils are activated by IFN-γ, which activates inducible nitric oxide synthase (iNOS) and the generation of NO. This molecule peroxidizes lipids and proteins, degrades the membrane, diffuses to the mitochondria of cardiocytes and triggers apoptotic or necrotic cell death. This is detrimental to mammals because of its high potential for tissue damage, and it has been regarded as a factor for myocarditis progression [78,79,82].

Finally, CD8+ T cells, a population that regulates protection against *T. cruzi* infection from the acute to the chronic phase [83], either by secreting cytokines such as TNF-α and IFN-γ or by releasing cytotoxic granules containing granzymes and perforins, are also capable of damaging the myocardium by cytotoxicity. In the acute phase, the absence of these cells results in an exacerbated parasitemia in a murine model; however, the presence of CD8+ T cells, especially in the chronic phase, is associated with areas of myocardial damage [84]. The presence of a perforin producing CD8+ T phenotype has been proven near areas of cardiac damage [85].

## 6. Fibrotic Mechanisms in Chagas Disease

Fibrosis in cardiac tissue is a significant increase in the volume of collagen. It gradually produces scar tissue, which affects the overall cardiac function in the severe manifestations of the chronic phase of Chagas disease [86,87]. Fibroblasts are responsible for the production of structural proteins that make up the extracellular matrix. This process, either constructive or reconstructive, favors cardiac remodeling, which is a progressive mechanism in response to acute or chronic damage, regardless of its etiology. Remodeling leads to changes in the heart size, shape, and function, and it is clinically associated with a poor prognosis in patients with heart failure [37,88].

Fibroblast activation is induced by various stimuli or by tissue injury; these trigger the production of transforming growth factor beta (TGF-β), epidermal growth factor (EGF), platelet-derived growth factor (PDGF), and fibroblast growth factor (FCF). Fibroblasts can also be activated by direct cell–cell communication and by contact with leukocytes through adhesion molecules such as the intercellular adhesion molecule-1 (ICAM-1) and the vascular cell adhesion molecule 1 (VCAM-1), or through reactive oxygen species or by complement [89]. This activation can lead to an excessive production and deposition of proteins in the extracellular matrix, a process known as fibrosis. In addition to being a source of collagenase, fibroblasts produce some cytokines (IL-1, IL-6) and enzymes such as matrix metalloproteinases (MMPs); this deposition of collagenase and fibers disrupts the connection between myocardial cells and compromises the structural and functional integrity of the heart. When these fibers are produced in excess, myocardial stiffness occurs. In turn, this condition shows disorders in parietal motility, with the consequent cavitary deformation (growth) and alteration in the diastolic and systolic functions of the heart [90].

Cardiac tissue fibrosis is accompanied by cellular inflammatory infiltrates characterized by the presence of macrophages, neutrophils, and eosinophils during the acute phase, and by lymphocytic infiltrates in the chronic phase. Acute or chronic fibrosis has been described in humans by immunohistochemistry in postmortem studies, pointing to the relevance of these proinflammatory cells and fibrotic accompaniment [91,92]. Cardiomyocytes have also been shown to actively participate in the inflammatory response by producing chemokines, cytokines, and NO. These factors can trigger leukocytes at-traction, and they have even been suggested to promote fibrosis by secreting fibro-blast-activating growth factors and cytokines [93]. It has been experimentally proven in primary cultures that soluble mediators synthesized by cardiomyocytes can regulate fibronectin synthesis [94].

Various works have studied in vitro the process of the extracellular matrix remodeling due to the presence of cytokines, mainly TGF-β, given that fibrosis is a characteristic process in Chagas chronicity. Other studies have reported that both TGF-β and IL-10 are major regulators of the Th1 response in the lapse preceding the onset of tissue damage, and decreasing the activation of phagocytic cells. On the other hand, TGF-β, IL-13, and IFN-γ are directly related to the severity of cardiac dysfunction due to degenerative fibrosis, which induces motility disorders and organ growth [94,95].

## 7. Conclusions

Altogether, the evasion of the immune response by *T. cruzi* and the proinflammatory immune response lead to autonomic heart dysfunction and microvascular disorders. They also activate autoimmune processes that result in the fibrosis that characterizes the chronic phase of the infection. These mechanisms, triggered by the presence of the parasite or its antigens, are responsible for the processes that underlie the immunopathogenesis of cardiac lesions in Chagas disease.

## Figures and Tables

**Table 1:** Main *T. cruzi* antigens that show molecular mimicry with vertebrate cell components.

Mammal Component	*T. cruzi* Antigens	Host	Ref.
β1-adrenergic receptors	P0 and P2β ribosomal proteins	Human	[52,53,54,55,56]
Smooth and striated muscle	150-kDa protein	Human, mouse	[57]
M2 cholinergic receptor	Not identified	Human	[58]
Myelin basic protein	*T. cruzi* soluble extract	Mouse	[59]
95-kDa myosin tail	*T. cruzi* cytoskeleton	Mouse	[60]
Cha antigen	SAPA, 36-kDa TENU2845	Mouse	[61]
Human cardiac myosin heavy chain	Cruzipain	Mouse	[19]
38-kDa heart antigen	R13 peptide of ribosomal protein P1, P2	Mouse	[62]
Calreticulin	Calreticulin	Human, mouse	[63]

**Table 2:** Evidence of autoimmune processes in Chagas disease.

Effects of Immunization with *T. cruzi* Antigens			
*T. cruzi* Antigen	Host	Effect	Ref.
Recombinant ribosomal protein P2β	Mouse	Alteration in ECG	[54]
R13 peptide of ribosomal protein P0	Mouse	Alteration in ECG	[58]
**Effects of passive transfer of antibodies or T cells from chronically infected *T. cruzi* hosts**			
**Immune effectors**	**Host**	**Effect**	**Ref.**
Splenocytes	Mouse	Focal myocarditis	[67]
T CD4+ cells	Mouse	Demyelination	[66]
Splenic T CD4+ cells	Mouse	Focal myocarditis	[68,69]
**Effects of immune tolerance induction with heart antigens**			
**Antigen**	**Host**	**Effect**	**Ref.**
Myosin	Mouse	Acute myocarditis, not modulated	[70,71]
Myosin-enriched heart lysate and homogenate	Mouse	Modulation of chronic myocarditis and fibrosis	[72]
